# Multicomponent Educational-Rehabilitation Approach in Rehabilitation of Patients After Stroke

**DOI:** 10.5152/eurasianjmed.2022.20330

**Published:** 2022-10-01

**Authors:** Alma Glinac, Osman Sinanović, Selma Sinanović

**Affiliations:** 1 University Clinical Center Tuzla, Ulica prof. dr. Ibre Pašića, Tuzla, Bosnia and Herzegovina; 2 Facullty of Education and Rehabilitation, University of Tuzla, Univerzitetska 1, Tuzla, Bosnia and Herzegovina; 3 Medical Faculty, Univeristy of Tuzla, Univerzitetska 1, Tuzla, Bosnia and Herzegovina; 4 Sarajevo School of Medcine, University of Sarajevo Scool of Science and Technology, Ilidža, Sarajevo, Bosnia and Herzegovina

**Keywords:** Educational-rehabilitation, multicomponent, stroke

## Abstract

Rehabilitation must be based on the individual needs and specific goals of the person and must be adapted to his abilities. According to the recommendation of the World Stroke Organization, the team involved in conducting rehabilitation should be multidisciplinary. One of the treatments that are applied within the multidisciplinary approach to a neurological patient is educational-rehabilitation treatment, which is multi-component in nature. Before starting educational-rehabilitation treatment, an educational-rehabilitation clinical assessment is necessary, which aims to detect difficulties caused by impairment; identify potentials and constraints in these areas; determine the specifics, course, and forecasts of difficulties; formulate clear treatment recommendations; form a watch list that will be available to all team members in the process of diagnosis, treatment, education, and to evaluate the effectiveness of treatment; and continuously monitor the ability and adaptive behavior of the person. Educational-rehabilitation clinical treatment includes treatment of cognitive abilities, treatment of motor skills, relaxation, treatment of adaptive skills, as well as informing the person about the disease and counseling.

This review focuses on some aspects of rehabilitation such as treatment of cognitive and motor disorders, treatment of adaptive skills, relaxation issues, and informing and counseling patients from the perspective of an educational rehabilitator with practical experiences in this area of rehabilitation.

**Main Points**

- An individual and holistic educational-rehabilitation approach in the rehabilitation of stroke patients, with a multidisciplinary team of experts, covers the complex motor and non-motor consequences of the disease, which affect the functional recovery and quality of life of the patient.
- Educational-rehabilitation treatment is based on a bio-psycho-social model in which biological, psychological, and social factors must be observed simultaneously, and rehabilitation must be focused on the person as a whole to achieve optimal physical, mental, and social potentials.

- Educational-rehabilitation clinical treatment includes treatment of cognitive abilities, treatment of motor skills, relaxation, treatment of adaptive skills, as well as informing the person about the disease and counseling.

## Introduction

According to the World Health Organization, rehabilitation is “a set of measures that help individuals, who have or are likely to have a disability, to achieve and maintain optimal functioning in interaction with their environment”.^1^

Rehabilitation must be based on the individual needs and specific goals of the person and must be adapted to his abilities.^2^ Treating a chronic patient does not only mean treating the disease but also all aspects that the disease affects, especially economic and social factors.^3^ Approaches to the treatment of patients should be holistic because without working on the cognitive, emotional, and physical aspects, the healing process is slowed down as well as the desired effect of medication.^4,5^

Stroke is the largest single cause of severe physical disability, and rehabilitation to reduce functional deficits is the most effective treatment.^6^ Functional recovery is based on the restitution of brain tissue and on the relearning of, and compensation for, lost functions.^7-9^ An important concept in rehabilitation is that of brain plasticity, which implies that it is possible to modulate or facilitate reorganization of cerebral processes by external inputs.^10^ Stroke rehabilitation is a process; its objectives are to prevent deterioration of function, improve function, and achieve the highest possible level of independence (physically, psychologically, socially, and financially) within the limits of the persistent stroke impairments. During this process, treatment and training are provided to stroke survivors to help them return to normal life. By regaining and relearning skills of everyday living through rehabilitation, many stroke survivors obtain greater independence in activities of daily living and improved functional capacity. Hallmarks of effective stroke rehabilitation practice include multidisciplinary/interdisciplinary team work, team work coordinated by regular meetings, goal-focused activities and individualized goals, emphasis on functional activities, involvement of patient and family in rehabilitation process, education provision to patients and families, and staff with specialized skills and interest in stroke.^11^

The World Stroke Organization has for the past 2 decades emphasized the importance of a multidisciplinary approach in providing recovery and rehabilitation services after stroke.^12^ The team involved in conducting rehabilitation should be multidisciplinary to include professionals such as a neurologist, physiatrist, nurses, physiotherapist, educational rehabilitator, speech therapist, neuropsychologist, occupational therapist, and social worker. Communication and coordination among these team members are paramount in maximizing the effectiveness and efficiency of rehabilitation.^13^

One of the treatments that are applied within the multidisciplinary approach to a neurological patient is educational-rehabilitation treatment (ERT), which is multicomponent in nature. Educational-rehabilitation treatment is based on a bio-psycho-social model in which biological, psychological, and social factors must be observed simultaneously, and rehabilitation must be focused on the person as a whole and on achieving optimal physical, mental, and social potentials.^14^ It is divided into stimulative treatment (encouraging the development of various abilities and skills), corrective treatment (based on direct action on endangered abilities or skills in order to alleviate or eliminate perceived difficulties), and compensatory treatment (based on the development of compensatory strategies). The methodology and organization of work should be flexible, guided by the characteristics and capabilities of the person and his environment, and not by some pre-given criteria for adapting to the requirements of the environment. The general approach to treatment is ecological, aimed at developing the potential and alleviating the limitations of the person in specific life circumstances and in light of the unique requirements of these circumstances.^15^

Before starting ERT, an educational-rehabilitation clinical assessment is necessary, which aims to: detect difficulties caused by impairment; identify potentials and constraints in these areas; determine the specifics, course, and forecasts of difficulties; formulate clear treatment recommendations; form a watch list that will be available to all team members in the process of diagnosis, treatment, education and to evaluate the effectiveness of treatment; and continuously monitor the ability and adaptive behavior of the person.

Educational-rehabilitation clinical treatment includes treatment of cognitive abilities, treatment of motor skills, relaxation, treatment of adaptive skills, as well as informing the person about the disease and counseling.

### Tretment of cognitive abilities

Cognitive functions are intellectual processes by which we become aware of something, perceive, and understand ideas. Therefore, they refer to the processes by which we receive and process information.^16^ The basic cognitive functions are attention, long-term memory, perception, while the higher cognitive functions are speech, language, decision-making, and executive functions.^17^ Frith and Dolan^18^ state that the difference between higher and lower cognitive functions is that the lower ones are automated and do not require special effort, while higher cognitive functions are under conscious control. However, as some higher cognitive functions, such as reading and understanding language, may become automated, the most important feature that distinguishes them is the fact that higher cognitive functions require more cognitive effort than lower cognitive functions. Cognitive training conducted by an educational rehabilitator has 4 levels.

The first level includes the treatment of cognitive functions that provide observation and input of new information, selection, and sorting of relevant information, as well as information maintenance, planning, organization, and control of activities. Namely, our sensory organs accept information from the environment and the body, and the process of sensory integration enables the brain to organize them and give them meaning. The brain can use only organized and integrated sensations for movement, learning, and behavior.^19^ Sensory stimulations of impaired central nervous system functions, with appropriate series of stimuli, chart a new path for collaterals of weakened or lost neurons and participate in their connection.^20^ The program of sensory stimulations includes visual, auditory, tactile, olfactory, gustatory, proprioceptive (sense of movement), and vestibular stimulation (sense of self-awareness—body position and movement in relation to gravity).

The ERT of visual functions include eye movement and focusing exercises, visual shape perception exercises (visual complementarity, background character discrimination, and visual organization), spatial relationships (spatial reasoning, spatial perception, and visual imagining), spatial orientations (laterality and direction), visual-motor integrations (motor response to visual stimulus and visual-motor coordination), visual memories (visual-spatial memory and visual-sequential memory), and speed of visual information processing (perceptual speed, automatism, and speed of motor responses).

Difficulties in visual perception cause difficulties in the area of higher visual functions and cognitive difficulties in terms of visual agnosia, alexia, prosopagnosia, achromatopsia.^21^ They also affect information processing speed, perceptual speed, as well as visual search (functional vision).^22^ Achtman et al^23^ proposed computer games as part of vision rehabilitation because of their impact on neuroplasticity of the visual system and visual learning. There is a large selection of vision exercises for impaired motility, binocular vision, and accommodation, for example, brock String, exercises with eccentric circles, Marsden ball, space fixator, McDonald field recognition exercise, Wayne Saccadic Fixator, and the like. Visual exercises primarily exercise visual functions but they also affect the functions of visual perception, such as visual-motor integration, visual-spatial organization, reaction speed, etc.^24^ Although the bulk of the voice of stroke rehabilitation is on the recovery of motor and communication functions, visual impairment is slowly beginning to receive the same amount of attention.^25^ The research indicates the need for vision rehabilitation to achieve maximum possible recovery in comprehensive rehabilitation and more successful performance of daily life activities.^26,27^ Vision rehabilitation has a positive effect on visual field width recovery,^28^ and visual field function can be compensated by increasing visual search field^29^ and increasing perception speed during visual search.^22^ The results confirmed the impact of vision rehabilitation on visual functioning in daily activities.^30^

There are numerous reliable sources on visual perception of patients with stroke.^25,27,28,32-39^

Difficulties in auditory information processing can be manifested by difficulty focusing on auditory stimuli, limited ability to listen continuously in the presence of noise, difficulty in understanding verbal information, limited ability to directly reproduce auditory information and follow orders of varying complexity, poor recognition and interpretation of different voices, reading and writing difficulties, poor speech, and lower academic achievement. Difficulties in the speed of processing auditory information can be reflected in understanding longer instructions and completing tasks related to reading and writing in a limited time (difficulties in understanding telephone voice messages, radio/television news, and movies). The treatment of auditory functions includes speed of auditory information processing, auditory discrimination, auditory sequencing, and auditory integration.

Treatment of sensory problems includes increasing tactile awareness, discrimination, and stereognosy through various tactile sensations (hard, soft, moist, dry, smooth, and rough). The program of kinesthetic perception includes differentiation of body scheme in gestural space, differentiation of facial mimicry, somatic stimulation of body and its parts, perception exercises in deep sensibility, and exercises for building and restoring body scheme. Several authors in their clinical research report that somatosensory training can lead to sensory improvement after stroke.^40-44^ Namely, after the somatosensory training, the somatosensory and functional changes of the upper extremities improve in terms of improving the ability to interpret bodily sensations and the feeling of control over one’s hand. Somatosensory training eases the use of the hand in daily life activities which is essential for continued brain and arm recovery.^45^

Attention is a means by which limited mental processes are directed to the information and cognitive processes that are most important at a given moment. The most common manifestations of attention difficulties are forgetting instructions, difficulties in organization of materials and activities, and difficulties in solving tasks. Disorders in the domain of visual and auditory attention can make it difficult to adopt and perform complex activities of everyday life, primarily due to difficulties in processing information or directing attention to relevant aspects of information. Educational-rehabilitation treatment contains 4 components of attention: focusing, maintaining, alternating, and sharing attention. The first group of exercises that are used are auditory attention range exercises (repetition of a series of numbers, letters, words, tones, or rhythms). The second group of exercises is focused on the development of strategies for processing and storing auditory verbal information (stimuli are given that can be grouped according to the functional, semantic, and phonological principles). In the treatment of the range of visual attention, exercises are used, which consist of a series of visual thymuses (objects, images, or movements) of increasing complexity, which a person should separate from a larger group and reproduce. Selective attention is practiced by applying tasks that require search, monitoring, rapid activation and inhibition of responses, and coordinated motor activity. In the domain of auditory attention, tasks are given in which the respondent should react (by raising his hand, moving a token, or taking notes) to the target word or sound in a series of auditory stimuli.

The second level of cognitive treatment includes memory treatment. Memory allows us to retain and find in our experience the information we use in the present.^46^ Memory difficulties can occur in the areas of encoding (initial organization of information for immediate reproduction or storage and later reproduction), consolidation (the process of transforming information from temporary, active process into permanent memory), and recollection (recalling stored information from long-term memory). The treatment should focus on metamemory, the creation, and the application of memory strategies. Exercises are of increasing complexity in different sensory modalities—auditory (verbal and nonverbal), visual (objects, illustrations, and shapes), and tactile-kinesthetic. In the field of auditory memory, exercises for direct and delayed reproduction of verbal and nonverbal information are applied. The skills that the client will stimulate through exercises are visual-auditory-kinesthetic strategies, associations, tracking orders, and recalling information. After memory-based cognitive rehabilitation, stroke patients reported fewer memory problems in everyday life immediately after treatment compared with control groups; however, there was no evidence that these beneficial effects persisted over a longer period.^47^

The third level of cognitive treatment refers to information processing and thinking that are responsible for the mental organization and reorganization of information: classification and organizational skills (separation, shaping, reasoning, combining, sorting, ranking, sequencing, and categorizing). The skills that the client will stimulate through exercises from this level are verbal and visual reasoning, thought organization, convergent reasoning, logic, comprehension, integration, reasoning, and problem-solving.

The last level includes the treatment of functions that ensure the expression and output of information (self-awareness, goal setting, self-control, flexible problem solving, and speech) and the treatment of difficulties in the field of academic skills (reading, writing, and mathematical skills).

Kim et al^48^ suggest that rehabilitation programs, for persons with stroke, should concentrate on increasing attention, concentration, information processing skills, memory, and patients’ judgmental ability to improve social cognition.

### Treatment of motor skills

Treatment of motor skills includes reeducation of motor skills, exercises for the development of basic and higher motor skills,^49^ and exercises aimed at the functional use of the hand and arm.

Motor reeducation is a multidimensional therapeutic approach in working with children and adults with motor disorders. Psychomotor reeducation is a specific field in educational and rehabilitation practice that focuses on the development of motor and motor skills, dexterity, balance, movement coordination and speed control, development of perceptual and gnostic abilities, and cognitive functions and contributes to the enrichment of sensory-motor and psychomotor experience.^50^ Reeducation of motor skills uses movement as a sensorimotor and psychomotor activity which summarizes the entire developmental course of the relationship to oneself and others that was formed during the sensorimotor and psychomotor relationship between a person and the world. In case of increased anxiety or other psychosomatic disorders, certain forms of motor reeducation significantly contribute to the reduction of physical and mental difficulties

The goal of reeducation is to stimulate, facilitate, or substitute dysfunctional cognitive mechanisms with more functional mechanisms to improve the client’s performance in those domains of behavior in which dysfunction or deficit is manifested.^51,52^ Motor reeducation treatment includes general reeducation exercises and specific reeducation exercises.

General motor reeducation exercises include massage, hydrotherapy, exercises with passive movements, exercises for defining the experience of body integrity, exercises for defining the experience of gestural space, exercises for independence of movement, exercises for equalizing muscle tone, observation exercises, stabilizing lateralization, exercises of experience and mastering the rhythm, exercises of experience, duration and orientation in time, exercises of coordination of movements, exercises for control of impulsivity, exercises for knowing the shape and weight, and exercises for noticing the presence of another. Exercises of specific reeducation of motor skills are directed toward individual and specific clinical pictures and are classified as follows: exercises for agraphia, exercises for alexia, exercises for acalculia and agnosia, exercises for apraxia, and exercises for motor speech disorders caused by neurological damage to nerves that participate in shaping voice with specific speech therapy treatment.^53^

Exercises for the development of basic motor abilities and skills are used to achieve better posture in different positions and activities, for better maintenance of balance at rest and movement, for more successful coordination of movements, for achieving greater precision of movement, and for more successful bimanual activities. The goals of treatment of higher motor abilities and skills are more precise execution of voluntary non-transitive and transitive movements, more harmonious organization of transitive movements in space and time, better organization of elements in 3-dimensional, 2-dimensional, and graphic space, and more successful coordination of movements with verbal or nonverbal orders. The following exercises are applied: exercises of melokinetic practice, ideomotor practice, ideational practice, construction exercises in 2-dimensional and 3-dimensional space, according to reproduction model or independent construction, exercises of graphomotor activities and visual-motor coordination (copying geometric shapes and drawings and drawing), and non-verbal movement regulation exercises.^15^ Upper extremity apraxia is a common stroke-related disorder that can reduce patients’ levels of independence in daily activities and increase their level of disability.^54^ The treatment of apraxia that has been studied in the literature,^55-57^ especially in stroke patients,^57,58^ proved effective. An adequate apraxia rehabilitation program can improve independence in daily life activities and accelerate the natural recovery process.^59^

The educational-rehabilitation program also includes motor skills exercises (locomotor activities like throwing, catching, and pushing; perceptual-motor activities important for the development of fine motor skills, coordination of the upper extremities, and visual-motor coordination; program for tone equalization and interdependence), exercises aimed at functional hand and arm use by performing coordinated activities required to move and handle objects using fists and hands (pulling, pushing, retrieving, manipulating, throwing, and grasping), and exercises aimed at improving fine motor skills by performing coordinated activities of handling objects, lifting, handling, and releasing by using 1 hand, fingers, and thumb (manipulating small objects).

Damage to the upper extremities after a stroke most often involves difficulties in moving and coordinating arm, hand, and fingers and often causes difficulties in performing daily activities such as eating, dressing, and washing. Dysfunction of the upper extremities, after stroke is the biggest reason for limitations in the functional use of hand in the daily activities of patients and their socialization.^60-62^ In stroke patients, the upper extremities recover more slowly functionally than the lower extremities.^63^

### Relaxation

Relaxation refers to a specific physiological state that is completely opposite to the way the body reacts when under stress or during a panic attack. Regular practice of deep relaxation can alleviate generalized anxiety and frequency and intensity of panic attacks, prevent stress accumulation, increase energy and productivity, improve concentration and memory, reduce insomnia and fatigue, reduce a number of psycho-somatic disorders^64^ and influence muscle relaxation, and improve concentration and attention.^65^ Some of the common methods for achieving a state of deep relaxation are: guided imagination, meditation, progressive muscle relaxation, self-training, yoga, abdominal breathing, soothing music, and the like.^64^ Guided imagination is the process of imagining objects, spaces, people, and situations, that is, stimulating visual, auditory, olfactory, taste, and proprioceptive sensations that are complementary to the sensory and aesthetic criteria of an individual. In this way, in addition to encouraging pleasure and satisfaction, more adequate imaginary systems of dealing with reality can be encouraged.^66^ Imagination also affects almost all important physiological mechanisms, such as respiration, pulse, blood pressure, sexual function, and the immune response, and can be used to relieve anxiety, depression, and stress symptoms.^67-69^ Breathing exercises are the basis of techniques used in different relaxation techniques with different therapeutic effects.^64,70-72^

### Treatment of adaptive skills

Adaptive behavior is a set of conceptual (decision-making, planning, and implementation of activities and verbal communication), social (reactions to social expectations/rules, interpersonal communication, self-esteem, and responsibility), and practical skills (activities of everyday life), which a person learned to function in everyday life. The level of adaptive behavior is conditioned by a number of personal and environmental factors, so when considering the approach to treatment, it is necessary to assess all potential factors that could be important for the development and modulation of various adaptive skills. As a result of impaired body structures and functions, after a stroke, a person may have difficulty performing basic activities of daily living which can lead to limitations in participation (e.g., in meaningful activities, community, family, work, social, and civic life),^73-75^ regardless of the severity of the stroke.^76^ Assessing and diagnosing adaptive skills are of great importance because adaptive skills help a person in performing everyday life activities.^77^ The treatment of adaptive skills aims to achieve an optimal level of independence in everyday life. In order for people with neurological diseases to have a better quality of life, it is very important to enable the acquisition of practical skills and to focus rehabilitation on strengthening independence and autonomy.

### Disease information and patient counseling

Information about the disease and counseling of patients are carried out with the aim of changing health behavior and raising the level of motivation of the individual in order to increase his desire for cooperation in rehabilitation and readiness to achieve goals aimed at successful rehabilitation. Motivation means the mental readiness of an individual to accept information. It depends on the relationship with the person conducting the education (information) and has the ability to convey the message in an understandable appropriate way. The most important for informing and educating patients is the interaction-communication relationship between educators and patients. At the very core of the relationship is empathy, as the educator’s ability to enjoy the role of the patient, to provide and enable support to the patient to make his or her own decision. Information and learning depend on the characteristics of the patient, such as his prior knowledge of the disease, resistance to the disease or its acceptance, and the support of the family and social environment. Positive transfer and countertransference are prerequisites for the success of the transmission of educational messages. Counseling as a 2-way relationship of patient training is essential for independent and successful solving of individual problems. It can be used in agreement on behavior change (in eating habits, smoking, and physical activity) and agreement on change of attitudes (toward illness, surgery, and social network).^78^

People react differently to the realization that they are seriously ill: they become disoriented, confused, their emotions numb, they distance themselves from the environment, and things that used to make sense become irrelevant. Therefore, support in coping with the disease is important, sometimes crucial for building a constructive response to the new situation. The patient is encouraged to think only about the next step, about small shifts, but special attention is paid to each achievement.^79^

Providing information to patients and caregivers improves their knowledge of stroke and increases patient satisfaction with some of the information they have received about stroke. An effect on reducing patients’ depression was also found. Providing information in a way that it involved patients and caregivers more actively, for example, when given more opportunities to ask questions, had a greater effect on patients’ mood than 1-time provision of information. There is little evidence that providing information has effects on independence or social activities.^80^

Since stroke carris with it two types of conseauences (motor and non motor) and rehabilitaion program should be focused equally on both areas, motor and non motor, respectively. Today, rehabilitation is more focused on the treatment of motor consequences, while non-motor ones are not given enough attention although they are an integral part of the clinical picture with a high prevalence and significantly incapacitate patients with stroke. The non-motor consequences of stroke, in addition to the cognitive ones we talked about, include speech, reading, writing, arithmetic disorders, neglect phenomena, anosognosia, agnosia, dysphagia, sleep disorders, as well as anxiety, depression, post-traumatic stress disorder, etc.^81^ All these disorders should be included in the comprehensive rehabilitation process, which implies the multidisciplinarity of the team, which must have a physiotherapist, speech therapist, neuropsychologist, and occupational therapist in addition to the educator-rehabilitator.

## Conclusion

Rehabilitation of patients with stroke should be focused on the person as a whole and on achieving optimal physical, mental, and social potentials. An individual and holistic educational-rehabilitation approach in the rehabilitation of stroke patients, with a multidisciplinary team of experts, covers the complex motor and non-motor consequences of the disease, which affect the functional recovery and quality of life of the patient.

In this review, the focus is only on some aspects of rehabilitation such as treatment of cognitive and motor disorders, treatment of adaptive skills, relaxation issues, and informing and counseling patients from the perspective of an educational rehabilitator with practical experiences in this area of rehabilitation.
